# Intranasal Immunization of Mice to Avoid Interference of Maternal Antibody against H5N1 Infection

**DOI:** 10.1371/journal.pone.0157041

**Published:** 2016-06-09

**Authors:** Fenghua Zhang, Bo Peng, Haiyan Chang, Ran Zhang, Fangguo Lu, Fuyan Wang, Fang Fang, Ze Chen

**Affiliations:** 1 College of Life Science, Hunan Normal University, Changsha, Hunan, China; 2 School of Medicine, Hunan Normal University, Changsha, Hunan, China; 3 School of Medicine, Hunan University of Chinese Medicine, Changsha, Hunan, China; 4 Department of Immunology, College of Basic Medical Sciences, Central South University, Changsha, Hunan, China; 5 Shanghai Institute of Biological Products, Shanghai, China; Shanghai Medical College, Fudan University, CHINA

## Abstract

Maternally-derived antibodies (MDAs) can protect offspring against influenza virus infection but may also inhibit active immune responses. To overcome MDA- mediated inhibition, active immunization of offspring with an inactivated H5N1 whole-virion vaccine under the influence of MDAs was explored in mice. Female mice were vaccinated twice via the intraperitoneal (IP) or intranasal (IN) route with the vaccine prior to mating. One week after birth, the offspring were immunized twice via the IP or IN route with the same vaccine and then challenged with a lethal dose of a highly homologous virus strain. The results showed that, no matter which immunization route (IP or IN) was used for mothers, the presence of MDAs severely interfered with the active immune response of the offspring when the offspring were immunized via the IP route. Only via the IN immunization route did the offspring overcome the MDA interference. These results suggest that intranasal immunization could be a suitable inoculation route for offspring to overcome MDA interference in the defense against highly pathogenic H5N1 virus infection. This study may provide references for human and animal vaccination to overcome MDA-induced inhibition.

## Introduction

Pregnant women and infants are usually the most susceptible populations to infection during influenza pandemics and seasonal epidemics, and influenza infection tends to result in more serious sequelae in these populations [1–3]. Infants under 6 months of age have been reported to have much higher morbidity and mortality rates than older babies during severe influenza seasons [4,5].

Vaccination is the best way to prevent influenza virus infection. However, the immune system of newborns is not mature enough to respond effectively to vaccination [6]. Additionally, no influenza vaccine is currently suitable for infants younger than 6 months [7]. Maternal immunization, which can provide the offspring with a high maternally-derived antibody (MDA) titer, may be a very good solution to this problem [8]. The inactivated influenza vaccine is recommended by the U.S. CDC for all pregnant women, especially those in the second or third trimester during influenza seasons or those with high risk conditions [9]. Vaccination protects not only the women but also their offspring from influenza. The passively delivered antibody (Ab) can delay the onset and decrease the severity of influenza disease in young infants [10,11]. However, in addition to the protective effect, MDAs have an inhibitory effect on the active immune response in the offspring. When the MDA titer is too low to provide protection but is sufficient to inhibit the active immune response, the infant is susceptible to influenza infection [12–14]. This inhibition often lasts for a long period of time and delays the vaccination of offspring against influenza. Thus, it is important to develop an effective immune strategy to overcome MDA interference. In our previous study, we recommended that different types of influenza vaccines (inactivated or DNA vaccine) or vaccines based on different virus antigens (HA or NA) be used for mothers and their offspring to effectively overcome the interference [15]. Despite this finding, the inactivated vaccine is currently the only type of licensed vaccine in most countries. Therefore, it would be better to adopt inactivated influenza vaccines for the active immunization of offspring to avoid MDA interference. Inactivated vaccines are administered parenterally in clinic and mainly induce serum IgG Abs. More and more studies have proved that intranasal (IN) immunization of inactivated influenza vaccines is effective in providing protection [16]. IN immunization induces not only systemic IgG but also local secretory-IgA Abs in the upper respiratory tract which can prevent the invasion of influenza viruses, and is therefore thought to be more potent than parenteral injection in influenza prevention [17]. Considering that the Abs transferred from mothers to offspring are primarily IgG class Abs, which would not infiltrate the nasal mucosa of the upper respiratory tract, IN immunization may be a means to avoid MDA-mediated inhibition.

Human infection with the H5N1 avian influenza virus was first confirmed in Hong Kong in 1997. To date, the highly pathogenic virus has infected hundreds of people worldwide with a high reported mortality rate of 53% [18]. The risk of an H5N1 pandemic still exists because humans generally lack immunity to the virus. For infants, it is particularly important to avoid MDA interference and establish active immune responses rapidly. In this study, infant BALB/c mice were inoculated with an inactivated H5N1 whole-virion vaccine in the presence of MDAs. Two inoculation routes (intraperitoneal (IP) and IN routes) were used to vaccinate the mothers and their offspring, and methods to overcome MDA interference were evaluated based on immune protection of the offspring.

## Materials and Methods

### Ethics statement

Six- to eight-week-old female BALB/c mice were purchased from the Center for Disease Control and Prevention in Hubei Province, China. The mice were bred in specific pathogen-free (SPF) animal houses under constant temperature and humidity conditions and a 12-hour light/12-hour dark cycle. They were grouped according to the experimental needs and kept in cages that had plenty of space for comfortable movement and easy access to food and water. All experiments involving animals were reviewed and approved by the Institutional Animal Care and Use Committee of Hunan Normal University (Permit Number: HNSD 2013–0046) in accordance with the animal ethics guidelines of the Chinese National Health and Medical Research Council (NHMRC).

### Virus and vaccine

The H5N1 influenza virus A/Chicken/Henan/12/2004(H5N1) used in this study was isolated from a chicken farm in Henan, China. The cloacal swab collected from a chicken was eluted using 2.0 ml phosphate-buffered saline (PBS) containing 0.1% bovine serum albumin (BSA), 4×10^6^ U/l penicillin G and 400 mg/l streptomycin sulfate, and then the elution fluid was sterilized by a 0.22-μm filter. After sterilization, the sample was inoculated into the allantoic cavities of 10-day-old SPF embryonated eggs (Beijing MERIAL Ltd., China). The allantoic fluid of the inoculated eggs was collected after incubation at 37°C for 48–72 h. The isolated virus was purified by three rounds of limiting dilution in embryonated chicken eggs, and then adapted in BALB/c mice as described previously [19,20]. The virus was stored at −70°C prior to use. All experiments with live H5N1 virus were performed under BSL-3 containment conditions.

The inactivated whole-virion vaccine was produced by the Shanghai Institute of Biological Products. The total protein concentration was measured using a BCA kit (Pierce, USA). The vaccine virus strain NIBRG-14, a reassortant between A/Vietnam/1194/2004(H5N1) and PR8, was provided by the National Institute for Biological Standards and Control (NIBSC), London, UK. The homologies of the hemagglutinin and neuraminidase proteins between the A/Chicken/Henan/12/2004(H5N1) and A/Vietnam/1194/2004(H5N1) virus strains were 98% and 97.3%, respectively (data in S1 Appendix).

### Immunization

The inactivated vaccine was diluted to 200 μl and 15 μl with PBS for the IP and IN administration routes, respectively.

Female BALB/c mice (aged 8 weeks) in the test groups were immunized twice at a 3-week interval with 1.0 μg of the inactivated vaccine through the IP or IN route. One week after the second immunization, the mice were bred with unimmunized male BALB/c mice aged 10–12 weeks. Approximately 15 days later, most of the females became pregnant and were separated from the males. Unimmunized female mice were used as the controls and treated with the same procedure. Pregnant female mice were fed water containing calcium and vitamins. After birth, the offspring were stayed with their mothers, and at 1 week of age, the offspring were grouped and immunized according to experimental requirements. The neonatal mice were immunized twice at a 3-week interval through the IP or IN route with the same inactivated vaccine. For the IP route, the offspring were injected with the vaccine at a dosage of 1.0 or 5.0 μg, and for the IN route, they were vaccinated at a dosage of 0.1, 1.0, or 5.0 μg. The offspring which were unimmunized were used as controls.

### Virus challenge

One week after the second immunization, the offspring were challenged with a lethal dose (40 LD_50_) of the mouse-adapted strain A/chicken/Henan/12/2004(H5N1) by nasal drip with 20 μl of the viral suspension. This infection caused rapid and widespread viral replication in the lungs and death of all unimmunized offspring within 7–10 days [15]. Survival and weight loss in the mice were monitored for 21 days. During this period, the mice were weighed and their health status was checked daily. When death appeared, the mice were observed 2–3 times a day. When the mice lost > 30% of their original weight or showed severe clinical signs, including gait instability and pre-comatose behavior, they were humanely euthanized via cervical dislocation after excess inhalation of chloroform to minimize or avoid animal suffering. At the end of the experiments, the surviving mice were humanely euthanized as described above.

### Sample collection

Blood samples were collected from the mothers through the tail vein 7 days after the second immunization and from the offspring before immunization, 21 days after the primary immunization and 7 days after the second immunization for the IgG Ab assay.

Three days after challenge, at least three mice in each group of offspring were humanely euthanized via cervical dislocation after chloroform inhalation and then exsanguinated from the heart with a syringe, which was performed as described in our previous study [15]. After blood collection, the mice were incised ventrally along the median line from the xiphoid process to the point of the chin. The trachea and lungs were removed and washed three times by injecting a total of 2 ml of PBS containing 0.1% BSA. The bronchoalveolar wash was used for virus titration after removing cellular debris by centrifugation.

After bronchoalveolar wash collection, the mouse head was removed and the lower jaw was cut off. A total of 1 ml of PBS containing 0.1% BSA was injected into the posterior opening of the nasopharynx three times to collect the outflow as the nasal wash. The nasal wash was centrifuged to remove cellular debris and used for the IgA Ab assay [21].

### Ab assays

The titers of IgG and IgA Abs produced against the inactivated whole-virion NIBRG-14 vaccine were measured by enzyme-linked immunosorbent assay (ELISA) as described in our previous study [15]. Briefly, the assay was performed using a 96-well microplate with successive reagents including the inactivated vaccine, serial twofold dilutions of sera from each group, goat anti-mouse IgG Ab (c-chain specific) or IgA Ab (α-chain specific) conjugated with biotin (Southern Biotechnology Associates, Inc. USA), streptavidin conjugated with alkaline phosphatase (Southern Biotechnology Associates, Inc. USA), and finally p-nitrophenyl-phosphate. The amount of chromogen produced was measured based on the absorbance at 414 nm and 405 nm by an ELISA reader (Labsystems Multiskan Ascent Autoreader, Finland). The antibody-positive cutoff value was set as the mean + 2SD of the negative control sera, and the Ab titer was expressed as the highest serum dilution showing a positive reaction.

### Virus titration

The bronchoalveolar washes were serially diluted 10-fold starting from a 1:10 dilution. Madin-Darby canine kidney cells were placed in a 24-well microplate, infected with each dilution, and incubated at 37°C in a carbon dioxide incubator for 48 h. Then, the cytopathic effects were examined. The virus titer of each specimen was expressed as the median tissue culture infection dose (TCID_50_) and was calculated by the Reed-Muench method. The virus titer in each experimental group was represented by the mean ± SD of the virus titers per ml of specimens from at least three mice in each group [15].

### Statistical analysis

The results of the experimental groups were evaluated by Student's *t*-test; the difference was considered significant when the *P* value was less than 0.05. The probability of survival was calculated using Fisher's exact test by comparing the survival rate of the offspring with MDAs to the offspring without MDAs or the mice in the negative control group. The survival patterns of the control and immunized offspring were graphed using Kaplan-Maier survival curves. The Log Rank test was used to analyze the survival rate data. Differences were considered significant at *P* < 0.05.

## Results

### Maternal antibodies induced by the inactivated vaccine through the IP or IN route were efficiently transferred to the offspring

Female BALB/c mice aged 8 weeks were divided into 3 groups (n = 10). Two groups were immunized twice with 1 μg of inactivated vaccine through the IP and IN routes, respectively, and the third group was unimmunized and used as a control. Seven days after the second immunization, sera were collected from the mice by tail vein bleeding. Then the mice were mated with unimmunized male BALB/c mice. Blood samples were collected from the offspring at 1 week of age. The sera of the mothers and offspring were used to determine the IgG Ab titers by ELISA. As shown in Table 1, mothers immunized with the inactivated vaccine through the IP or IN route had high IgG Ab titers after the booster, and their offspring had Ab titers nearly as high as their mothers. This result indicated that the Abs induced by immunization of the mothers through either the IP or IN route were efficiently transferred to the offspring.

**Table 1:** Antibody titers in mother mice after immunization and in their offspring before immunization ^a^.

Immunization route	Dose (μg)	Serum IgG Ab titers (log_2_)^b^	
		Mothers (after immunization)	Offspring (before immunization)
IN	1.0	20.25±0.5	19.75±0.5
IP	1.0	19.75±0.5	19.5±0.6

^a^ Female mice were immunized twice at a three week interval with 1 μg of inactivated vaccine through the IP or IN route. Blood samples were collected by tail vein bleeding 7 days after the second immunization. The mice were mated with unimmunized male mice. Serum samples were collected from the offspring at 1 week of age (before immunization). The IgG Ab titers were measured by ELISA.

^b^ Values represent the mean ± S.D. of each group.

### Protection of the offspring when both mothers and offspring were immunized via the IP route

Female BALB/c mice aged 8 weeks were divided into 2 groups (n = 20). One group of mice was immunized twice with 1 μg of the inactivated vaccine through the IP route and the second group was not immunized. The offspring with MDAs (i.e., born to immunized mothers) were divided into 3 groups (n = 15). Two groups of offspring aged 1 week were immunized with 1 μg or 5 μg of the inactivated vaccine through the IP route and the third group was unimmunized and used as the MDA+ control group. The offspring without MDAs (i.e., born to unimmunized mothers) were also grouped and handled similarly as those with MDAs, and among them, the unimmunized offspring were used as the MDA- control (negative control). Seven days after the second immunization, all offspring were challenged with a lethal dose (40 LD_50_) of influenza virus. Three days after virus challenge, at least three mice from each group were sacrificed for lung virus titration. The rest of the offspring were observed for 3 weeks to record the survival rate and body weight change.

**Fig 1:**
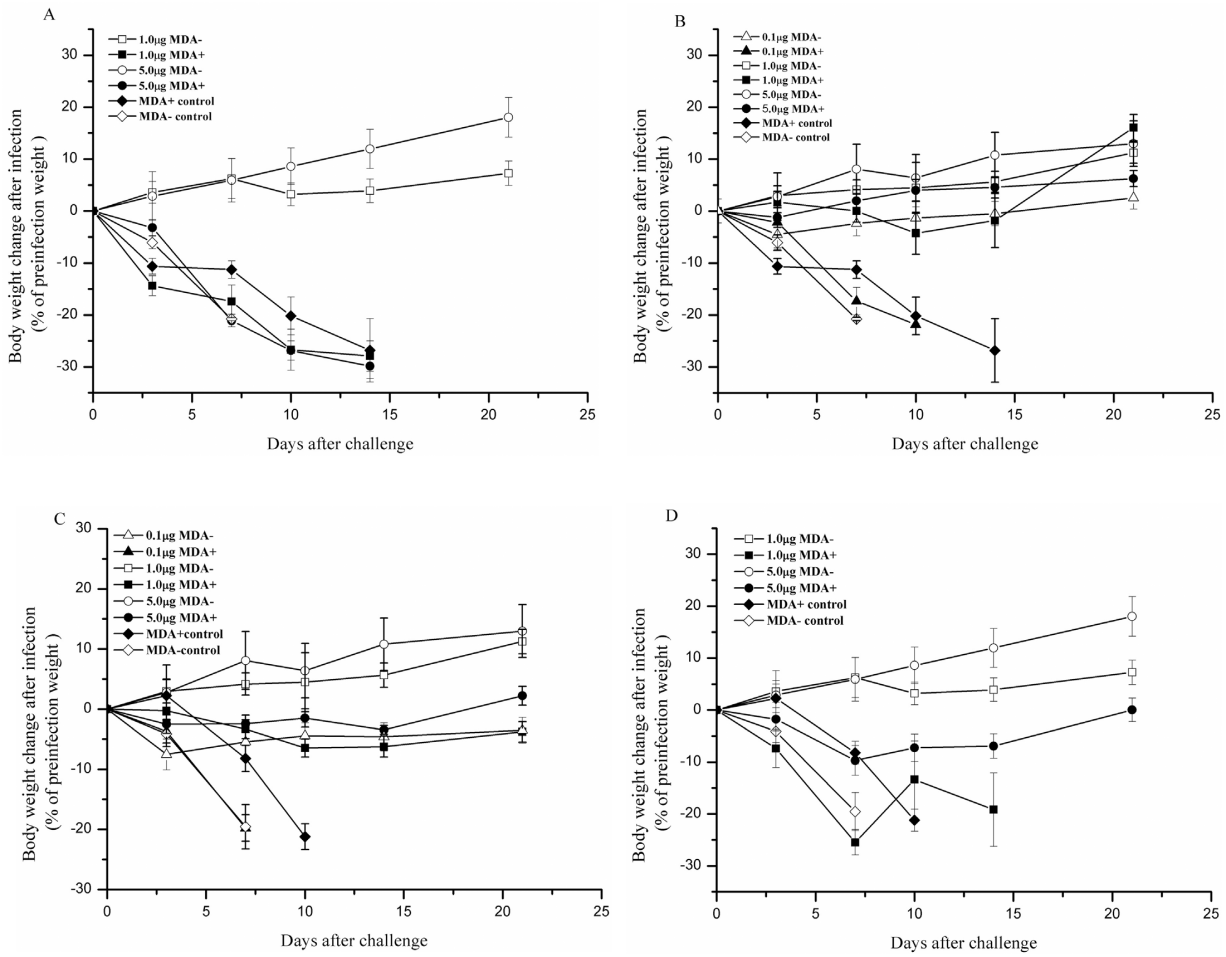
Body weight changes in the offspring within 21 days after the lethal virus challenge. A). Both mothers and offspring were immunized with the inactivated vaccine through the IP route. B). Mothers were immunized through the IP route and offspring through the IN route. C). Both mothers and offspring were immunized through the IN route. D). Mothers were immunized through the IN route and offspring through the IP route. Data points represent the means ± SD of each group of mice. “MDA- control” indicates that the offspring in this group had no MDAs and were unimmunized; “1μg MDA+” indicates that the offspring in this group had MDAs and were immunized with 1 μg of the vaccine.

**Fig 2:**
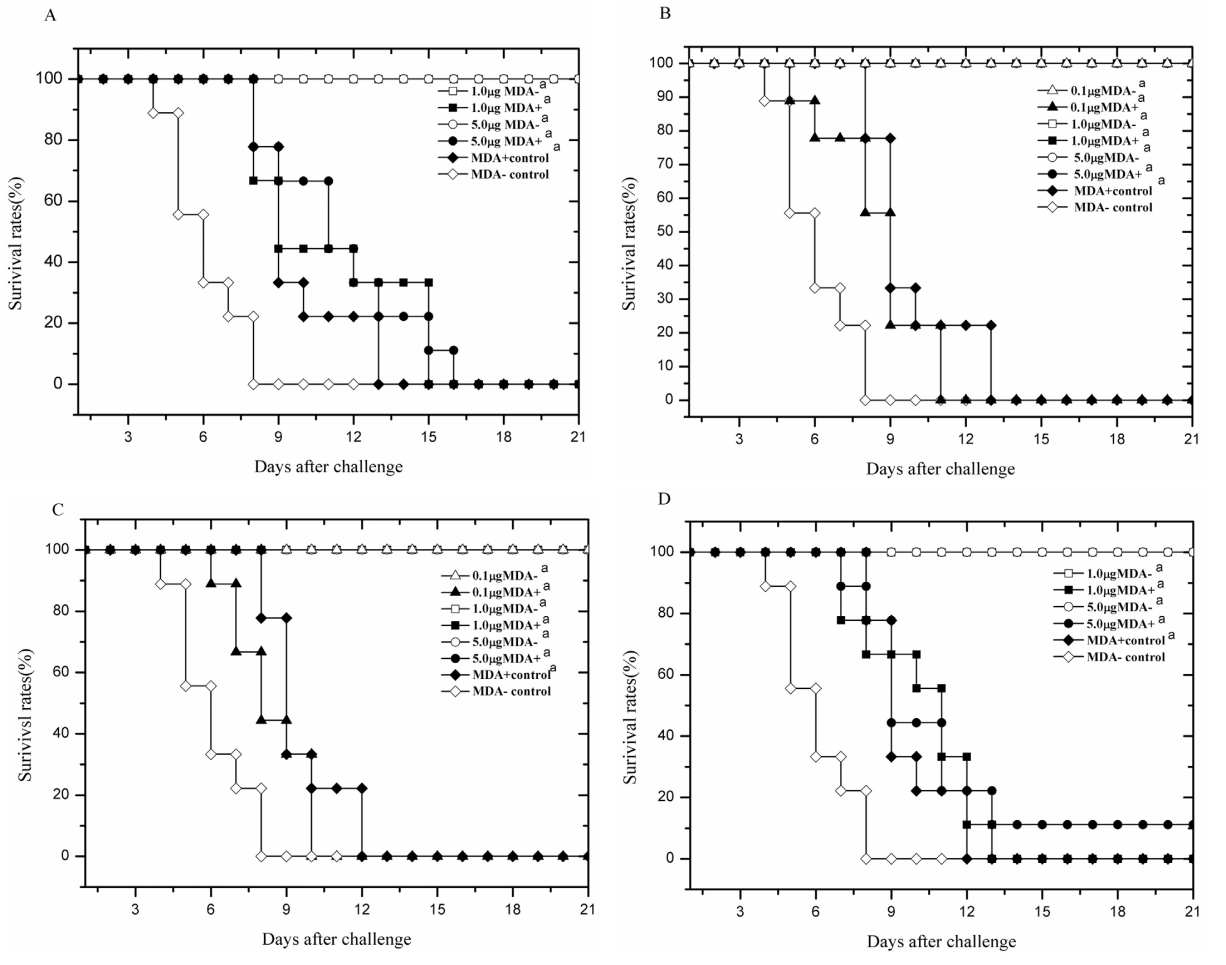
Survival rates of offspring over time after lethal virus challenge. A). Both mothers and offspring were immunized with the inactivated vaccine through the IP route. B). Mothers were immunized through the IP route and offspring through the IN route. C). Both mothers and offspring were immunized through the IN route. D). Mothers were immunized through the IN route and offspring through the IP route. Data points represent the means ± SD of each group of mice. “MDA- control” indicates that the offspring in this group had no MDAs and were unimmunized; “1μg MDA+” indicates that the offspring in this group had MDAs and were immunized with 1 μg of the vaccine. ^a^Significant differences (*p* < 0.05) compared with the MDA- control determined by the Log Rank test.

The results are shown in Table 2. The offspring with MDAs all died (0% survival rate) after the lethal virus challenge regardless of whether they were immunized. In contrast, the immunized offspring without MDAs all survived the challenge. The survival rate (100%) was significantly different from the survival rates in the negative control group and in the offspring with MDAs. The offspring with MDAs exhibited severe weight loss before death, but their time of death was delayed compared with the offspring in the negative control group in which all mice died within 8 days post-challenge. The offspring without MDAs immunized with 1 μg of the vaccine exhibited less weight loss and restored weights within 10 days after the virus challenge. When the mice were immunized with 5 μg of the vaccine, they lost nearly no weight and even gained weight after the challenge (Figs 1A and 2A). The results also showed that the residual lung virus titers in the offspring with MDAs were high, although they were significantly lower than those in the offspring of the negative controls. No residual lung virus titers were detected after challenge in the offspring without MDAs immunized with either 1 μg or 5 μg of the vaccine (Table 2).

**Table 2:** Antibody responses and protection of offspring when both mothers and offspring were immunized via the IP route^a^.

Mothers	Vaccine dosage for offspring (μg)	Serum IgG Ab titers in offspring (log_2_)^b^		Lung virus titers (log_10_ TCID_50_)^b^	Survival offspring/tested offspring
		Prime	Boost		
Immunized	1.0	11.50±0.58^d^	12.33±0.58^c^, ^d^	14.60±0.55^c^, ^d^	0/9^c^
	5.0	11.00±0.00^c^, ^d^	14.25±0.50^c^, ^d^	13.38±0.28^c^, ^d^	0/9^c^
	unimmunized	11.75±0.50^d^	10.30±0.50^d^	13.83±0.44^d^	0/9
Unimmunized	1.0	11.25±0.50^d^	16.50±0.58^d^	Undetected^d^	9/9^d^
	5.0	13.50±0.58^d^	18.50±0.58^d^	Undetected^d^	9/9^d^
	unimmunized	<1	<1	19.5±0.17	0/9

^a^ Female mice were immunized twice at a 3 week interval with the inactivated vaccine through the IP route. The offspring were also immunized through the IP route at 1 and 4 weeks of age. Serum samples were collected from the offspring 3 weeks after the primary immunization and 1 week after the booster. Serum IgG Ab titers were measured by ELISA. One week after the booster, the offspring were challenged with a lethal dose (40 LD_50_) of A/Chicken/Henan/12/2004 (H5N1). Lungs were removed from three mice in each group 3 days after challenge for virus titration by the standard MDCK assay. Survival rates of the mice were determined 3 weeks after the challenge.

^b^ Values represent the mean ± S.D. of each group.

^c^ Significant difference (*p* <0.05) compared with the same dosage group of offspring without MDAs.

^d^ Significant difference (*p* <0.05) compared with the negative control.

Sera were collected from the offspring by tail vein bleeding 3 weeks after the primary immunization and 1 week after the booster and used to determine the IgG Ab titers by ELISA. The results are shown in Table 2. In the offspring immunized with 1 μg of the inactivated vaccine, the Ab titers in the offspring with MDAs were equal to those in the offspring without MDAs after the primary immunization. After the booster, the Ab titers in the offspring with MDAs rose slightly but were significantly lower than those in the offspring without MDAs. In the offspring immunized with 5 μg of inactivated vaccine, the Ab titers in the offspring with MDAs were significantly lower than those in the offspring without MDAs after both the primary and booster immunizations. Additionally, the MDA titers in the offspring in the MDA+ control group (unimmunized) declined over time and were lower than those in the immunized offspring with or without MDAs.

The above results indicated that maternal immunization with the inactivated vaccine through the IP route inhibited the active immune response in the offspring vaccinated via the same route. Moreover, even a high dose of vaccine could not overcome the inhibition in the offspring.

### Protection of the offspring when mothers were immunized via the IP route and the offspring via the IN route

Female BALB/c mice were grouped and handled similarly as described above. The offspring with MDAs were divided into 4 groups (n = 15). Three groups of offspring aged 1 week were immunized with 0.1 μg, 1 μg and 5 μg of inactivated vaccine through the IN route. The fourth group of offspring was unimmunized and used as the MDA+ control. The offspring without MDAs were handled similarly as those with MDAs, and the unimmunized offspring were used as MDA- control.

The results showed that the survival rates of the offspring with MDAs in the control group and the 0.1 μg immunization group were 0% after virus challenge, whereas the survival rates were 100% in the 1 μg and 5 μg immunization groups. The survival rates of the offspring without MDAs immunized with 0.1 μg, 1 μg or 5 μg of the inactivated vaccine were all 100% (Table 3). The weight changes and survival of mice over time after virus challenge are shown in Figs 1B and 2B. The offspring with MDAs had significantly lower lung virus titers than the offspring in the negative control group regardless of whether they were immunized, and the lung virus titers in the 1 μg and 5 μg immunization groups were very low. In the offspring without MDAs, the lung virus titers in the 0.1 μg immunization group were significantly lower than the titers in the negative control group, and no residual lung virus titers were detected in the 1 μg and 5 μg immunization groups (Table 3).

**Table 3:** Antibody responses and protection of offspring when mothers were immunized via the IP route and their offspring via the IN route^a^.

Mothers	Vaccine dosage for offspring (μg)	Ab titers in offspring (log_2_)^b^			Lung virus titers (log_10_ TCID_50_) ^b^	Survival offspring/tested offspring
		Serum IgG Ab		sIgA Ab		
		Prime	Boost	Boost		
Immunized	0.1	11.33±0.58	10.00±0.00^c^,^d^	1.68±0.58	14.20±0.34^c^,^d^	0/9^c^
	1.0	11.75±0.50	14.33±0.58^c^,^d^	2.00±0.00	2.72±0.54^c^,^d^	9/9 ^d^
	5.0	13.33±0.58	14.00±1.0^c^,^d^	2.67±0.58	2.67±0.34^c^,^d^	9/9^d^
	unimmunized	11.75±0.50	10.30±0.50	Undetected	13.83±0.44^d^	0/9
Unimmunized	0.1	13.50±0.58	15.00±0.82^d^	1.50±0.50	5.00±1.00^d^	9/9^d^
	1.0	13.75±0.50	17.33±0.58^d^	2.33±0.58	Undetected^d^	9/9^d^
	5.0	15.75+0.50	19.33±0.58^d^	3.33±0.58	Undetected^d^	9/9^d^
	unimmunized	<1	<1	<1	18.33±0.58	0/9

^a^ Female mice were immunized twice at a 3 week interval with the inactivated vaccine through the IP route. The offspring were immunized through the IN route at 1 and 4 weeks of age. One week after the booster, the offspring were challenged with a lethal dose (40 LD_50_) of A/Chicken/Henan/12/2004 (H5N1). Serum samples were collected from the offspring 3 weeks after the primary immunization and 1 week after the booster, and nasal washes were collected 3 days after the challenge. Serum IgG and sIgA Ab titers were measured by ELISA. Lungs were removed from three mice in each group 3 days after challenge for virus titration by the standard MDCK assay. Survival rates of the mice were determined 3 weeks after the challenge.

^b^ Values represent the mean ± S.D. of each group.

^c^ Significant difference (*p* <0.05) compared with the same dosage group of offspring without MDAs.

^d^ Significant difference (*p* <0.05) compared with the negative control.

The serum IgG Ab titers are shown in Table 3. In the offspring with MDAs, the Ab titers after the booster decreased in the 0.1 μg immunization group but increased in both the 1 μg and 5 μg groups compared with the titers after primary immunization. However, all of the titers were significantly lower than those in the offspring without MDAs at the same dosages. This finding indicated that the MDAs inhibited the production of IgG Abs in the offspring immunized via the IN route and that the inhibition was weakened by increasing the vaccine dosage. Three days after virus challenge, nasal washes were collected from the offspring for the detection of sIgA Ab titers by ELISA. The sIgA Abs were detected in the nasal washes of immunized offspring with or without MDAs. Higher sIgA Ab titers were correlated with higher immunization dosages. Additionally, no significant difference in sIgA Ab titers was detected between offspring with and without MDAs that received the same dosage (Table 3). This finding suggested that MDAs did not influence sIgA Ab production.

The results indicated that IN immunization of the offspring overcame MDA interference with the active immune response when the mother mice were immunized with the inactivated vaccine through the IP route.

### Protection of the offspring when both mothers and offspring were immunized via the IN route

Female BALB/c mice were grouped and handled similarly as described above except that they were immunized via the IN route, and their offspring were grouped and immunized via the IN route, as is described above.

The results are shown in Table 4. When IN administration was used for both mothers and offspring, the survival rate after virus challenge in the 0.1 μg immunization group was 0%, which was the same as the survival rates in the MDA+ and MDA- control groups. In the 1 μg and 5 μg immunization groups, the survival rate was 100%. In addition, for the offspring without MDAs, the survival rates in the 0.1 μg, 1 μg and 5 μg immunization groups were all 100%. The body weight changes and survival of mice over time after virus challenge are shown in Figs 1C and 2C. The lung virus titers in the offspring with MDAs in the 0.1 μg, 1 μg and 5 μg immunization groups were significantly lower than those in the offspring of the negative control group but were obviously higher than those in the offspring without MDAs at the same dosages.

**Table 4:** Antibody responses and protection of offspring when mothers were immunized via the IN route and their offspring via the IN route^a^.

Mothers	Vaccine dosage for offspring (μg)	Ab titer in offspring (log_2_)^b^			Lung virus titers (log_10_ TCID_50_)^b^	Survival offspring/tested offspring
		Serum IgG Ab		sIgA Ab		
		Prime	Boost	Boost		
Immunized	0.1	13.00±0.81	12.00±0.00^c^,^d^	1.75±0.50	13.99±0.72^c^,^d^	0/9
	1.0	11.75±0.50	14.00±1.0^c^,^d^	2.00±0.81	3.00±0.27^c^,^d^	9/9^d^
	5.0	11.00±0.00	15.33±0.58^c^,^d^	3.250±0.50	3.21±0.60^c^,^d^	9/9^d^
	unimmunized	12.25±0.50	10.00±1.00^d^	Undetected	13.72±0.26^d^	0/9
Unimmunized	0.1	13.75±0.50	15.00±0.00^d^	1.50±0.50	4.67±0.58^d^	9/9^d^
	1.0	13.75±0.50	17.33±0.58^d^	2.33±0.58	Undetected	9/9^d^
	5.0	15.75±0.50	19.33±0.58^d^	3.33±0.58	Undetected	9/9^d^
	unimmunized	<1	<1	<1	18.89±0.2	0/9

^a^ Female mice were immunized twice at a 3 week interval with the inactivated vaccine through the IN route. The offspring were also immunized through the IN route at 1 and 4 weeks of age. One week after the booster, the offspring were challenged with a lethal dose of A/Chicken/Henan/12/2004 (H5N1) (40 LD_50_). Serum samples were collected from the offspring 3 weeks after the primary immunization and 1 week after the booster, and nasal washes were collected 3 days after the challenge. Serum IgG and nasal wash sIgA Ab titers were measured by ELISA. Lungs were removed from three mice in each group 3 days after challenge for virus titration by the standard MDCK assay. Survival rates of the mice were determined 3 weeks after the challenge.

^b^ Values represent the mean ± S.D. of each group.

^c^ Significant difference (*p* <0.05) compared with the same dosage group of offspring without MDAs.

^d^ Significant difference (*p* <0.05) compared with the negative control.

The IgG Ab titers in the sera of offspring were determined 3 weeks after the primary immunization and 1 week after the booster as shown in Table 4. In the offspring with MDAs, the Ab titers after the booster were decreased in the 0.1 μg immunization group but increased in both the 1 μg and 5 μg groups compared with the titers after primary immunization. However, all of the titers were significantly lower than those in the offspring without MDAs at the same dosages. Three days after virus challenge, the nasal mucosal sIgA Ab titers were measured by ELISA. The sIgA Abs were detected in the nasal washes of immunized offspring with or without MDAs. Their titers were positively related to the immunization dosage. There were no significant differences in the sIgA Ab titers between the offspring with and without MDAs at the same dosages (Table 4). The results suggested that the MDAs from mothers vaccinated via the IN route inhibited the production of serum IgG Abs in the offspring, although the inhibition became weak as the immunization dosage increased. Moreover, IN immunization of the offspring helped to overcome MDA interference with the active immune response.

### Protection of the offspring when mothers were immunized via the IN route and the offspring via the IP route

Female BALB/c mice were grouped and handled as described above and immunized via the IN route, and their offspring were handled the same as described above and immunized via the IP route.

The results are shown in Table 5. For the offspring with MDAs, the survival rates after virus challenge in the 1 μg and 5 μg immunization groups were 0% and 11.1%, respectively. The residual lung virus titers were high but were significantly lower than those in the offspring of the negative control group. For the offspring without MDAs, the survival rates in the 1 μg and 5 μg immunization groups were all 100%, and no residual virus titers were detected in the lungs. The body weight loss and survival of the mice over time after virus challenge are shown in Figs 1D and 2D. Sera were collected from the offspring and used to determine the IgG Ab titers by ELISA 3 weeks after the primary immunization and 1 week after the booster. The Ab titers in the offspring with MDAs were increased after the booster but were still significantly lower than those in the offspring without MDAs at the same immunization dosages. The results suggested that IP immunization of the offspring with the inactivated vaccine even at a high dose could not overcome MDA interference from mothers vaccinated via the IN route.

**Table 5:** Antibody responses and protection of offspring when mothers were immunized via the IN route and their offspring via the IP route^a^.

Mothers	Vaccine dosage for offspring (μg)	Serum IgG Ab titers in offspring (log_2_)^b^		Lung virus titers (log_10_ TCID_50_) ^b^	Survival offspring/tested offspring
		Prime	Boost		
Immunized	1.0	11.00±0.00	12.67±0.58^c^,^d^	13.57±0.83^c^,^d^	0/9^c^
	5.0	10.67±0.58	13.00±0.00^c^,^d^	12.78±0.69^c^,^d^	1/9^c^
	unimmunized	12.25±0.50	10.00±1.00^d^	13.72±0.26^d^	0/9
Unimmunized	1.0	11.50±0.58	16.50±0.58	Undetected	9/9^d^
	5.0	13.50±0.58	18.50±0.58	Undetected	9/9^d^
	unimmunized	<1	<1	18.33±0.33	0/9

^a^ Female mice were immunized twice at a 3 week interval with the inactivated vaccine through the IN route. The offspring were immunized through the IP route at 1 and 4 weeks of age. One week after the booster, the offspring were challenged with a lethal dose (40 LD_50_) of A/Chicken/Henan/12/2004 (H5N1). Serum samples were collected from the offspring 3 weeks after the primary immunization and 1 week after the booster. Serum IgG antibody titers were measured by ELISA. Lungs were removed from at least three mice in each group 3 days after challenge for virus titration by the standard MDCK assay. Survival rates of the mice were determined 3 weeks after the challenge.

^b^ Values represent the mean ± S.D. of each group.

^c^ Significant difference (*p* <0.05) compared with the same dosage group of offspring without MDAs.

^d^ Significant difference (*p* <0.05) compared with the negative control

## Discussion

At present, the H5N1 virus is considered to be the most likely causative agent of a new influenza pandemic because humans generally lack pre-existing immunity to this virus [22,23]. The H5N1 virus has a high pathogenicity and fatality rate and poses a great threat, especially to pregnant women and young infants. Maternal immunization can protect not only mothers but also early stage infants from H5N1 influenza virus infection. In our previous study, we demonstrated that maternal immunization with the inactivated H5N1 whole-virion vaccine protected mouse offspring aged 1–4 weeks against lethal challenge with a homologous virus and that the MDAs in the offspring were detectable 13 weeks after birth [24]. However, the residual MDAs interfered with active immunization of the offspring. When the MDA titers were too low to provide protection but were sufficient to inhibit the active immune responses, the offspring were susceptible to influenza infection [12–14]. The phenomenon of MDA interference affects vaccination against many infectious diseases [25–28].

Many studies concerning the MDA interference have showed that the inhibitory effect of MDAs primarily impacts the humoral immune response and not the cellular immune response in the offspring [29,30]. In a review by Siegrist CA [30], four mechanisms were reported to contribute to MDA interference during the active immunization of infants: 1) neutralization of the administered vaccines; 2) inhibition of infant B cell activation by Fcγ receptor-mediated signals; 3) effective elimination of MDA-coated antigen via Fc-dependent phagocytosis; and 4) epitope masking by MDAs that prevents the antigen binding by infant B cells. In our experiments, the influence of MDAs on the vaccination of mouse offspring against influenza H5N1 infection was explored. We showed that maternal Abs were efficiently transferred to the offspring regardless of the immunization route (IP or IN) used for the mother mice (Table 1). Moreover, the offspring with MDAs had significantly lower IgG Ab titers than those without MDAs regardless of the immunization route (IP or IN) used for the offspring, indicating that MDAs interfered with IgG Ab production in the offspring. However, the interference was weakened when the immunization dosage for the offspring was increased (Tables 2–5). The result demonstrated that the interference effect of MDAs on the active immunization of offspring was inversely proportional to the amount of MDAs in the offspring, which was identical to our previous study [15]. Increasing the immunization dosage for the offspring may reduce the amount of MDAs and thus alleviate their interference with active immunization.

Apart from increasing the vaccine dosage, other methods have been applied to avoid MDA interference, such as postponing vaccination [31], shortening the interval between the prime and the boost [32], and inoculating mothers and their babies with different types of vaccines [15]. The immunization route may also impact MDA interference. Different vaccine administration routes induce different immune responses. Chiu et al. demonstrated that only 40% the influenza vaccine dosage required for an intramuscular injection was needed to reach a similar immune response level when the inactivated influenza vaccine was injected intracutaneously [33]. Because the Abs transferred from mothers to offspring are primarily IgG class Abs, they may predominantly influence the IgG Ab response rather than local IgA Ab activity in the offspring. Thus, mucosal immunization could be attempted to avoid MDA-mediated inhibition. At present, the only type of influenza vaccine that is used clinically and delivered mucosally is the live attenuated influenza vaccine (LAIV). The LAIV is administered intranasally and induces strong sIgA Ab responses at the natural site of virus entry, thereby effectively preventing influenza infection. However, this type of vaccine has some limitations and is not widely used [34,35]. For instance, LAIV is recommended only for people 5–49 years of age. Inactivated vaccines are the licensed influenza vaccines worldwide and are administered by intramuscular injection. Nowadays, an increasing number of studies have confirmed the efficacy of IN administration with inactivated influenza vaccines [36–39]. Takada et al. [37] demonstrated that mice intranasally immunized with several subtypes of inactivated whole-virion vaccines alone (without adjuvant) obtained protection against an H5N1 subtype virus. Okamoto et al. [36] demonstrated that similar levels of protection were achieved by IN immunization of mice with an inactivated influenza whole-virion vaccine alone and with a split-virion vaccine plus mucosal adjuvants. Based on these experimental results and due to its many obvious superiorities [40], the mucosal route might be a potential immunization method for inactivated influenza vaccines.

In our present experiments, the immune effects of the IP and IN routes were compared between the offspring with and without MDAs. The offspring were vaccinated with inactivated H5N1 influenza vaccine at 1 week of age because the MDA titers were high at this age (only slightly lower than those in their mothers) and therefore MDA interference was obvious. As a control, offspring without MDAs obtained a 100% survival rate after lethal virus challenge whether they were immunized through the IP or IN route and with a higher (5 μg) or lower (1 μg) vaccine dosage. However, the immune effects for the offspring with MDAs were different. The offspring immunized via the IP route with a 1 μg or 5 μg vaccine dosage all died after challenge (Tables 2 and 5), whereas the offspring immunized via the IN route all survived the challenge even at the 1 μg vaccine dosage (Tables 3 and 4). Both the IP and IN routes induced a similar level of systematic IgG Abs in the offspring with MDAs, but the IN route simultaneously produced mucosal sIgA Abs. Thus, the sIgA Abs played a very important role in overcoming the interference mediated by MDAs.

Just as expected, the result in our experiments showed that MDAs only inhibited the production of IgG Abs in the offspring and did not affect sIgA production. The offspring with MDAs had approximately the same sIgA levels as the offspring without MDAs, and the sIgA Ab titer increased concomitantly with the increase in the vaccination dosage (Tables 3 and 4). These results are similar to those reported by Kitikoon et al, who found that the amount of MDAs in piglets infected with a sublethal dose of influenza virus had almost no influence on the local IgA responses [41]. However, some studies on the swine LAIV reported that MDAs interfered with the mucosal antibody response [42]. The divergent results might be due to the use of different experimental designs, animal models and vaccine types [17,43]. Additionally, many studies found that MDAs only inhibited the humoral immune response and had no effect on the cellular immune response [44,45]. We did not detect an influence on the cellular immune response after IN vaccination, but in our previous study we confirmed that cell-mediated immunity induced by IN administration of a subunit vaccine based on the viral internal protein NP or M1 played an important role in the protection of mice against a lethal virus challenge [46,47]. Therefore, the cellular immune response might offer protection in offspring with MDA interference.

At last, we found that, at the same vaccine dose, the sIgA levels were approximately the same in the offspring with MDAs and without MDAs, while IgG Ab titers were different between them, leading to different protective abilities (Tables 3 and 4). This indicated that both the amounts of IgG Abs in serum and sIgA Abs contributed to defending the virus. Serum IgG Abs are sufficient to prevent influenza infection of the lung and only highly protective against homologous virus infection, while local sIgA Abs are the major factor for preventing influenza infection in the upper respiratory tract and can provide cross-protection against influenza viruses [17,43]. Therefore, IN inoculation of inactivated vaccines, which induces not only IgG but also sIgA Abs, is more effective in overcoming MDA interference. In addition, IN administration has a series of advantages, including easily acting out, needle-free administration, no special training and fewer cross infections, which makes it a suitable route for infants and young children in vaccination of influenza vaccines.

To summarize, our results suggested that the MDAs induced by the administration of the H5N1 whole-virion vaccine through the IP or IN route were efficiently transferred to the offspring and interfered with the active immune responses. IN immunization of the offspring with the inactivated vaccine could overcome the MDA interference and provide effective protection even when the MDA titers were high in the offspring. This study may provide insights into vaccination of humans and animals against influenza virus infection in early life.

## Supporting Information

S1 Appendix Genbank accession numbers of HA and NA genes from the two influenza virus strains in our study. (DOCX)
