# Supplementary material for: Intranasal Immunization of Mice to Avoid Interference of Maternal Antibody against H5N1 Infection
Source: PLoS One. 2016 Jun 9;11(6):e0157041. doi: 10.1371/journal.pone.0157041 (PMC4900595; doi:10.1371/journal.pone.0157041)
Supplement: S1 Appendix — (DOCX) [file pone.0157041.s001.docx]

**S1 Appendix. Genbank accession numbers of HA and NA genes from the two influenza virus strains in our study.**

**A/Chicken/Henan/12/2004(H5N1)**
HA: GenBank: AY950232.1
http://www.ncbi.nlm.nih.gov/nuccore/AY950232.1

NA: GenBank: AY950246.1
http://www.ncbi.nlm.nih.gov/nuccore/ay950246.1

**A/Vietnam/1194/2004(H5N1)**
HA: GenBank: EF541402.1
http://www.ncbi.nlm.nih.gov/nuccore/EF541402.1

NA: GenBank: EF541466.1
http://www.ncbi.nlm.nih.gov/nuccore/EF541466.1
